# The Transcription Coregulator RIP140 Inhibits Cancer Cell Proliferation by Targeting the Pentose Phosphate Pathway

**DOI:** 10.3390/ijms23137419

**Published:** 2022-07-04

**Authors:** Valentin Jacquier, Delphine Gitenay, Vincent Cavaillès, Catherine Teyssier

**Affiliations:** 1 IRCM, Institut de Recherche en Cancérologie de Montpellier, INSERM U1194, University of Montpellier, 34298 Montpellier, France; valentinjacquier1993@gmail.com (V.J.); vincent.cavailles@inserm.fr (V.C.); 2 IRMB, University of Montpellier, INSERM, 34295 Montpellier, France; delphine.gitenay@inserm.fr

**Keywords:** breast cancer, pentose phosphate pathway, G6PD, transcription, RIP140

## Abstract

Cancer cells switch their metabolism toward glucose metabolism to sustain their uncontrolled proliferation. Consequently, glycolytic intermediates are diverted into the pentose phosphate pathway (PPP) to produce macromolecules necessary for cell growth. The transcription regulator RIP140 controls glucose metabolism in tumor cells, but its role in cancer-associated reprogramming of cell metabolism remains poorly understood. Here, we show that, in human breast cancer cells and mouse embryonic fibroblasts, RIP140 inhibits the expression of the gene-encoding G6PD, the first enzyme of the PPP. RIP140 deficiency increases G6PD activity as well as the level of NADPH, a reducing cofactor essential for macromolecule synthesis. Moreover, G6PD knock-down inhibits the gain of proliferation observed when RIP140 expression is reduced. Importantly, RIP140-deficient cells are more sensitive to G6PD inhibition in cell proliferation assays and tumor growth experiments. Altogether, this study describes a novel role for RIP140 in regulating G6PD levels, which links its effect on breast cancer cell proliferation to metabolic rewiring.

## 1. Introduction

The pentose phosphate pathway (PPP) is an alternate metabolic pathway to glycolysis. Upon cell uptake through its transporters, glucose is converted by hexokinase into glucose-6-phosphate, which can then be metabolized through the PPP. The PPP is broadly separated into two steps: the oxidative branch that generates nicotinamide-adenine dinucleotide phosphate (NADPH) and the non-oxidative branch that interconverts sugars providing intermediates for the nucleotide biosynthesis or the glycolytic pathway [1]. Depending on the needs of the cell, NADPH maintains cellular redox homeostasis or is used for anabolic reactions. Hence, PPP could promote cancer progression and resistance to treatment [2].

Glucose 6-phosphate dehydrogenase (G6PD) sets the pace of the PPP, and its activity determines the first committed step of the pathway. Upregulation of G6PD level or activity is observed in many cancers, including pancreatic, cervical, gastric, colonic, and invasive breast carcinomas [2,3]. Several signaling pathways are responsible for promoting G6PD expression or activity in cancer cells. In colon cancer cells, p53 interacts with G6PD and inhibits its activity independently of transcription [4]. However, despite the crucial role of the PPP in tumorigenesis, its regulation is not fully understood.

The transcription coregulator RIP140 is involved in the tuning of many transcription factors, mostly preventing their transactivation ability through the recruitment of histone deacetylases [5]. RIP140 exerts critical roles in tumorigenesis [6]. It inhibits the proliferation of intestinal cells and regulates the APC/β-catenin pathway in colorectal cancers. High expression of RIP140 correlates with good prognosis of colorectal cancer patients [7,8]. On the other hand, low RIP140 expression is associated with basal-like breast cancer subtypes [9]. Ambivalent effects of RIP140 on breast tumor cell proliferation have been reported with both positive [10,11,12] and negative [9,13,14] effects.

Interestingly, our recent work showed that RIP140 inhibits glucose-dependent proliferation of breast cancer cells by blocking glycolysis [14]. We then wondered whether RIP140 could also be involved in the transcriptional regulation of PPP genes in breast cancer. The present study identifies G6PD as an RIP140 target gene necessary for the proliferative advantage of RIP140-deficient cells. RIP140 inhibits the expression of G6PD in breast cancer cells and in immortalized or transformed mouse embryonic fibroblasts (MEFs) from RIP140 knock-out (RIPKO) mice. RIP140 deficiency increases G6PD activity and, as a correlate, the NADPH/NADP+ ratio. Interestingly, down-regulating G6PD expression or inhibiting its enzymatic activity results in a decrease in cell proliferation of RIP140-deficient cells. Altogether, our data demonstrate that RIP140 participates in the transcriptional regulation of PPP in breast cancer, thus reinforcing its impact on glucose metabolism in tumor cells.

## 2. Results

### 2.1. RIP140 Inhibits G6PD Gene Expression at the Transcriptional Level

**Figure 1:**
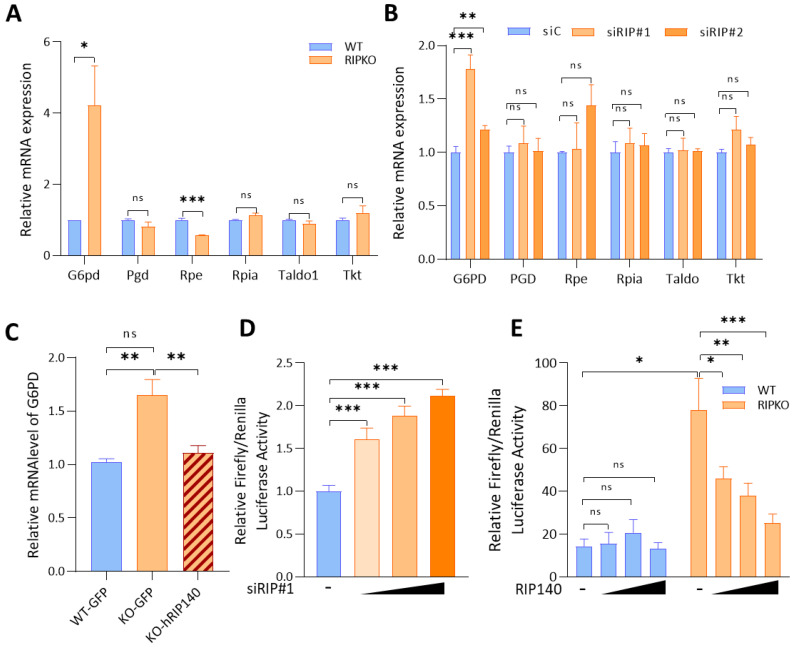
**RIP140 inhibits G6PD gene expression at the transcriptional level.** (**A**,**B**) mRNA expression of the indicated genes was quantified by RT-qPCR in MEF WT or RIP140 knock-out (RIPKO) (values are normalized to WT samples) (**A**) and in MDA-MB-436 transfected with control siRNA (siC) or RIP140 siRNAs (siRIP#1; siRIP#2). Values are normalized to control siRNA samples (**B**). Name of the genes: G6PD: glucose-6-phosphate dehydrogenase; PGD: 6-phospho-gluconate dehydrogenase; RPE: ribulose-5-phosphate-3-epimerase; RPIA: ribose 5-phosphate isomerase A; TALDO1: transaldolase 1; TKT: transketolase. (**C**) G6pd mRNA expression level was assessed by RT-qPCR in MEF WT or RIP140 knock-out (RIPKO) overexpressing GFP or human RIP140 (hRIP140). The values are normalized to WT-GFP samples. (**D**) Luciferase activity assays were conducted in MDA-MB-436 cells transfected with a G6PD-Luc reporter gene, the luciferase reporter TK-Renilla, and in increasing concentrations of RIP140 siRNA (siRIP#1). Luciferase values were normalized to the Renilla luciferase control and to the values of samples transfected with control siRNA (−). (**E**) Luciferase activity assay in MEF WT or RIPKO transfected with a G6PD-Luc reporter gene, the luciferase reporter TK-Renilla and increasing concentrations of a RIP140-expressing plasmid. Luciferase values were normalized to the Renilla luciferase control. All experiments were conducted at least three times. Values are means ± SEM; * *p* < 0.05, ** *p* < 0.01, *** *p* < 0.001, ns = not significant.

To evaluate the role of RIP140 in the regulation of the PPP, we first compared the mRNA level of PPP enzymes found in immortalized wild-type (WT) mouse embryonic fibroblasts (MEFs) with the level found in MEFs that were depleted for the RIP140 gene (RIPKO). We observed a significant increase in the G6PD mRNA level and a significant decrease in the mRNA level of the ribulose-5-phosphate 3-epimerase (Rpe), one enzyme of the non-oxidative branch, in RIPKO MEFs (Figure 1A and Supplementary Figure S1A).

We downregulated the expression of RIP140 in two breast cancer cell lines, MDA-MB-436 and MCF7, using specific RIP140 siRNA (Supplementary Figure S1B). Analysing PPP gene mRNA level using RT-qPCR revealed that G6PD was the only PPP gene deregulated after RIP140 down-regulation in both breast cancer cells. RIP140 silencing did not affect the Rpe mRNA level in breast cancer cell lines. Different regulators involved in Rpe transcriptions may be required, specifically in RIPKO fibroblasts, but not in epithelial cancer cells (Figure 1B and Supplementary Figure S1C).

Furthermore, rescuing RIP140 expression in RIPKO MEFs downregulated G6PD mRNA levels (Figure 1C and Supplementary Figure S1D).

We then further deciphered the effects of RIP140 on G6PD expression. Performing luciferase reporter assays using the G6PD promoter showed that RIP140 knock-down increased the luciferase activity of this reporter in MDA-MB436 cells and in RIPKO MEFs (Figure 1D,E). Moreover, RIP140 overexpression decreased the luciferase activity driven by the G6PD promoter (Figure 1D), confirming the transcriptional inhibition of G6PD expression by RIP140.

### 2.2. RIP140 Deficiency Increases G6PD Activity

**Figure 2:**
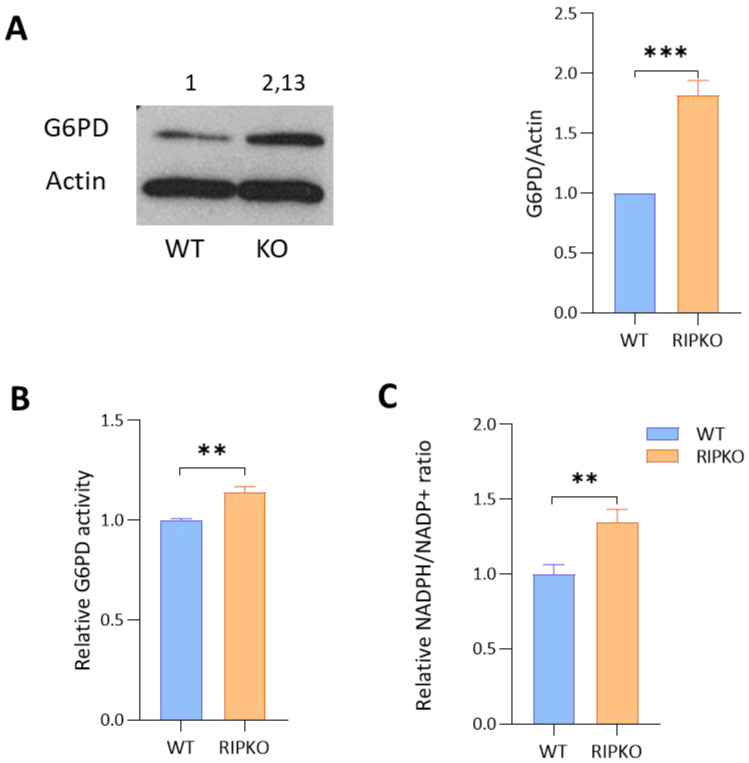
**RIP140 deficiency increases G6PD activity.** (**A**) The expression of G6PD and actin were assessed by Western blot analysis in WT or RIP140 knock-out (RIPKO) MEFs (left panel). Quantification was performed using Image J software on three independent experiments. Values were normalized to the actin signal and to WT samples (right panel). (**B**) The enzymatic activity of G6PD was quantified in WT or RIPKO MEFs using the G6PD assay kit (Sigma). Values are normalized to cell number and to WT samples. (**C**) The NADPH/NADP^+^ ratio was quantified in WT or RIPKO MEFs using the Promega NADP/NADPH-Glo Assay. Data were normalized to protein concentration and to WT samples. All experiments were conducted at least three times. Values are means ± SEM; ** *p* < 0.01, *** *p* < 0.001.

We then wanted to confirm the regulation of G6PD expression by RIP140 at the protein level. Western blot analysis showed that the amount of G6PD protein was increased in the absence of RIP140 in MEFs (Figure 2A and Supplementary Figure S2A) and in MCF7 cells after RIP140 silencing (Supplementary Figure S2C). Using enzymatic assays, we confirmed that G6PD activity was higher in RIPKO than in WT MEFs (Figure 2B and Supplementary Figure S2B). As a correlate of this increased G6PD activity, the NADPH/NADP+ ratio was higher in RIPKO cells than in control cells (Figure 2C). Altogether, these results confirmed that RIP140 deficiency led to an increase in G6PD activity in MEFs.

### 2.3. G6PD Is Required for the Proliferative Advantage of RIP140-Deficient Cells

**Figure 3:**
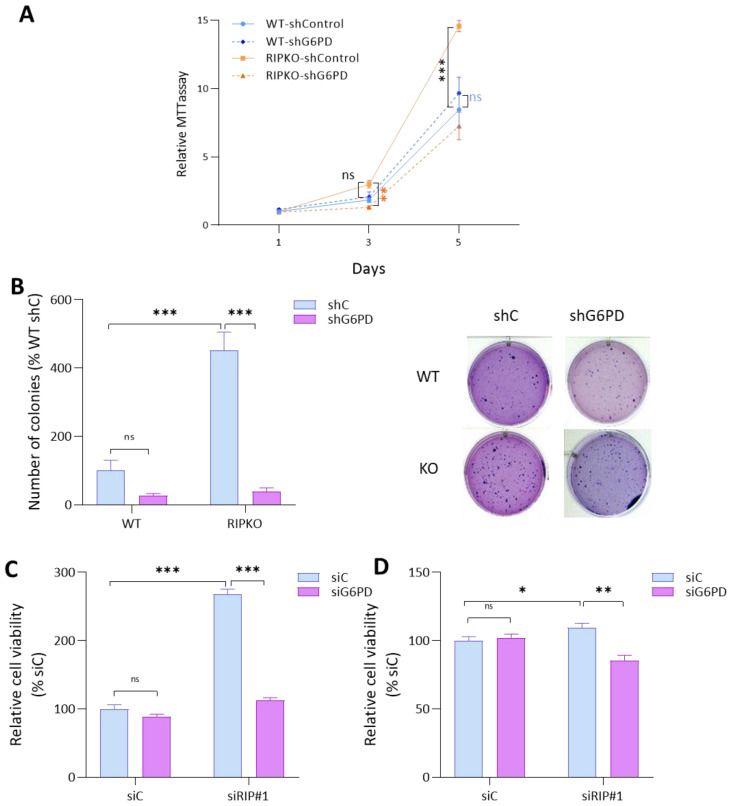
**G6PD is required for the proliferative advantage of RIP140-deficient cells.** (**A**) 3-(4,5-dimethylthiazol-2-yl)-2,5-diphenyltetrazolium bromide (MTT) assay in WT or RIPKO MEFs transfected with shRNA control or shG6PD expression lentivirus. The data are normalized to day 1 values for each shRNA and to WT control shRNA. Blue asterisks correspond to differences between WT samples, orange ones to differences between KO samples, and black ones to differences between WT and KO samples. (**B**) Number of colonies from a colony formation in soft-agar assay of transformed MEF WT or RIPKO transfected with shRNA control or G6PD expression lentivirus. The data are expressed as percentages of WT shControl samples (shC) (left panel). Pictures of colonies in soft agar stained by crystal violet (right panel). (**C**,**D**) Cell viability assessed by crystal violet staining of MDA-MB-436 cells (**C**) and MCF7 cells (**D**) transfected with RIP140 siRNA (siRIP) combined with G6PD siRNA. The data are normalized to day 1 values for each siRNA and then to control siRNA (siC) samples representing the growth advantage of siRIP140 transfected cells over siC cells. All experiments were conducted at least three times. Values are means ± SEM; * *p* < 0.05, ** *p* < 0.01, *** *p* < 0.001, ns = not significant.

We previously described that RIP140 deficiency resulted in a cell growth increase [9,13,14]. We then tested whether G6PD was required by RIP140-deficient cells to proliferate at a higher rate. Knocking down G6pd mRNA with small hairpin RNA (shRNA) in MEFs (Supplementary Figure S3A) either abrogated the growth advantage of RIPKO MEFs, as shown by MTT (Figure 3A), or significantly reduced it, as shown in colony formation assays (Figure 3B). We could repeat this result by down-regulating RIP140 and G6PD expression concomitantly by siRNA in the breast cancer cell lines MDA-MB-436 and MCF7 (Figure 3C). Altogether, these data demonstrate that G6PD overexpression is required for the increase in cell proliferation observed in RIP140-deficient cells.

### 2.4. RIP140 Deficiency Sensitizes Cells to G6PD Inhibition

**Figure 4:**
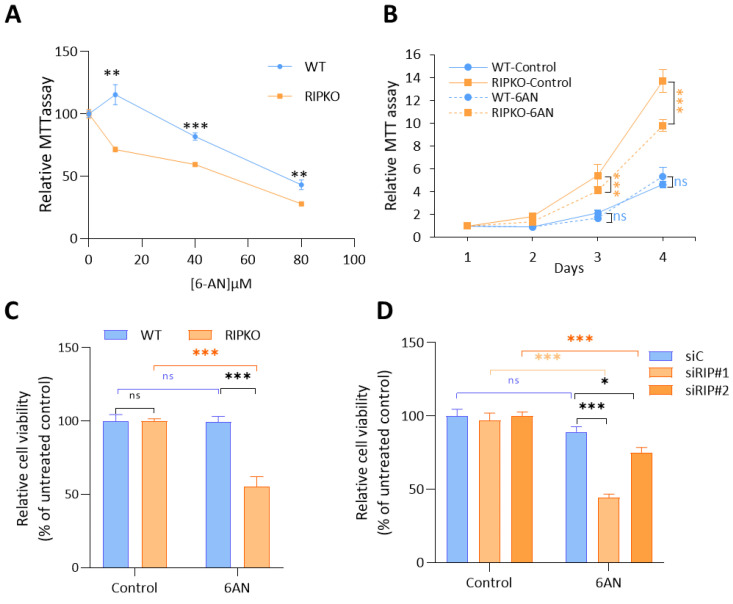

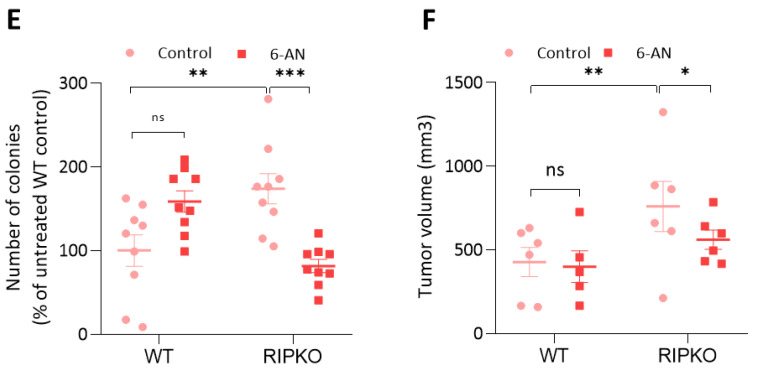
**RIP140 deficiency sensitizes cells to G6PD inhibition.** (**A**) MTT assay in WT or RIPKO MEFs after 4 days of increasing doses of 6-aminonicotinamide (6AN) at 10, 40 and 80 µM. Values are normalized to day 1 for MEF WT or RIPKO and to control samples expressed as 100%. (**B**) MTT assay in WT or RIPKO MEFs treated with 20 µM of 6AN. (**C**) Cell viability assessed by crystal violet staining of transformed WT or RIPKO MEFs treated for 7 days with 6AN (20 µM). Values are normalized to that of untreated MEFs. (**D**) Cell viability assessed by crystal violet staining of MDA-MB-436 cells transfected with control siRNA (siC) or RIP140 siRNA (siRIP#1, siRIP#2) and treated for 7 days with 6AN (20 µM). Values are normalized to that of untreated control siRNA. (**E**) Number of colonies from a colony formation in soft-agar assay of transformed WT or RIPKO MEFs transfected with shRNA control or G6PD expression lentivirus and treated for 4 weeks with 6AN (20 µM). The data are expressed as percentages of WT untreated control samples. (**F**) Tumor volume of transformed MEF WT or RIPKO xenografted in nude mice (*n* = 6) after eighteen days of 6AN administrated intra-peritoneally every other day (0.1 mg/g). All experiments were conducted at least three times. Values are means ± SEM; * *p* < 0.05, ** *p* < 0.01, *** *p* < 0.001, ns = not significant. The colors of asterisks correspond to the same differences as in Figure 3A.

To further demonstrate the importance of G6PD activity in RIP140-deficient cells, we treated immortalized MEFs with various doses of 6-aminonicotinamide (6AN), a specific inhibitor of G6PD enzymatic activity. We found that increasing 6AN doses reduced the growth of RIPKO MEF to a higher extent than WT cells, as shown by MTT assays (Figure 4A). To better define the 6-AN concentration to use in breast cancer cells, we performed a growth inhibition curve using the IncuCyte^®^ Live Cell Analysis Imaging System (Supplementary Figure S4C). As 20 µM of 6-AN inhibited cell proliferation about 70% in both breast cancer cell lines, we fixed the concentration of 6AN at 20 µM. This allowed us to show that the drug significantly impacted the growth of RIPKO MEF, whereas it did not affect that of WT cells at this chosen concentration (Figure 4B). The same results were obtained using transformed MEFs in a cell viability assay (Figure 4C and Supplementary Figure S4A) and after down-regulating RIP140 by siRNA in the breast cancer cell lines MDA-MB-436 and MCF7 (Figure 4D and Supplementary Figure S4E). Furthermore, performing a colony formation assay showed that 6AN treatment did not affect the growth of WT colonies, whereas it did reduce that of RIPKO colonies (Figure 4E and Supplementary Figure S4D).

To confirm these results in vivo, we xenografted transformed MEFs into nude mice that received 6AN intraperitoneally. We showed that the volume of WT tumors did not change upon 6AN treatment, while the drug specifically reduced the volume of RIPKO tumors (Figure 4F). Altogether, these results demonstrated that RIP140 deficiency sensitizes cancer cells to G6PD inhibition.

## 3. Discussion

The transcriptional coregulator RIP140 is a key player in metabolic homeostasis, regulating lipid metabolism and mitochondrial oxidative phosphorylation in adipocytes and muscles [15]. However, its role in cancer metabolism remains poorly described. Our previous work has shown that RIP140 impinges breast cancer cell proliferation by blocking glycolysis through the inhibition of the glucose transporter GLUT3 expression [14].

Here, we demonstrate that RIP140 also inhibits the expression of G6PD, the first enzyme of PPP. This metabolic pathway plays a critical role in cancer cell survival and growth by producing pentose phosphate for nucleic acid synthesis and providing NADPH. This reducing agent is necessary for the synthesis of macromolecules, and is essential for tumor cells to fight against oxidative stress [1]. Previous studies indicate that oncoproteins, such as Ras, Akt, and mTOR, regulate the PPP in tumor cells to increase cell survival and proliferation [16]. Therefore, the PPP flux regulatory network represents an important metabolic adaptation in a number of environmental settings in human malignancies, including cancer. Metabolic adaptation is a characteristic of tumor cells that confers essential benefits in terms of growth. Our findings show that RIP140 negatively regulates G6PD activity, at least in transformed MEFs, thus strengthening its link with tumor cell metabolic reprogramming.

The overexpression of G6PD has been described in many types of cancer with poor outcomes. Increased expression of G6PD is a predictive indicator of high risk of relapse and metastasis in patients with breast cancer [17,18]. Because G6PD overexpression creates favorable conditions for cancer cells to thrive, it is of importance to better understand its regulation. G6PD regulatory networks are complex in cancer cells, and multiple cis/trans elements regulate G6PD expression. At the transcriptional level, some transcription factors have been described to regulate G6PD expression in various cancers [19]. For instance, NF-κB and pSTAT3 synergistically drive G6PD overexpression and facilitate sensitivity to G6PD inhibition in clear-cell renal cell carcinoma [20]. The transcription factor YY1 activates the expression of G6PD in colorectal, endometrial, and hepatic cancer cells [21].

Estradiol is also known to regulate G6PD expression and activity in breast cancer [22]. RIP140 was initially identified through its action as an agonist-dependent coregulator of estrogen receptors [23]. Here, we identify RIP140 as a novel transcriptional regulator of G6PD expression in breast cancer cells. Our data show that RIP140 inhibits the expression of G6PD at the mRNA and protein levels. Interestingly, RIP140 deficiency augments the stimulatory effect of estradiol on G6PD expression in MCF7 breast cancer cells, suggesting that RIP140 impairs estradiol-induced G6PD expression at least in ER+ breast cancer cells (Supplementary Figure S5A).

Our previous study revealed an interaction between RIP140 and the hypoxia-inducible factors (HIFs) [14], also known to regulate G6PD expression [24]. Of note, hypoxia-induced G6PD expression was abolished in RIPKO MEFs (Supplementary Figure S5B), suggesting that HIF-RIP140 interplay may be involved in the regulation of G6PD by hypoxia. Deciphering the molecular mechanism responsible for G6PD expression regulation, i.e., finding the transcription factor(s) mediating RIP140 effects in ER+ and ER− breast cancers, requires further investigations, and could improve our knowledge of transcriptional control of the PPP.

RIP140 deficiency, at least in MEFs, leads to increased G6PD activity accompanied with a high NADPH/NADP^+^ ratio. Thus, the PPP could be more active upon RIP140 deficiency, producing more NADPH, which is crucial to suppress intracellular ROS, and could favor cell proliferation. Indeed, RIP140 deficiency increased cell proliferation and tumor growth in breast cancer cells and MEFs, respectively. Interestingly, the down-regulation of G6PD expression reduced the growth advantage of RIP140-deficient cells. Moreover, G6PD inhibition by a specific inhibitor resulted in a reduction in the growth of RIP140-KO MEF tumors and of RIP140-deficient breast cancer cells. Indeed, similar results were found in the literature. For instance, high-G6PD-expressing bladder cancer cells displayed higher sensitivity to 6-AN compared with lower-G6PD-expressing cells [25]. In addition, 6-AN induced apoptosis in primary AML cells with higher levels of G6PD, but did not affect the survival of normal hematopoietic progenitor cells [26]. In fact, it is conceivable that cells with high G6PD expression are highly dependent on its activity for survival and that inhibition of G6PD, even at low concentrations, has a strong impact on the survival of such cells. Altogether, these results confirm the rationale for targeting G6PD activity in cancer. Indeed, targeting G6PD with inhibitors has been shown to be a promising strategy for the treatment of cancer [19]. Our results suggest that targeting G6PD with inhibitors in breast cancer patients that have a low level of RIP140 expression might be more efficient. On the other hand, the down-regulation of G6PD expression could also reverse the proliferative effect due to RIP140 deficiency in some cases (Figure 3A,D), suggesting that the level of G6PD might impinge on the effect of RIP140 on cell proliferation, which is known to be versatile in different cell types [10,14].

Altogether, our data reveal a new regulator of the oxidative branch of the PPP through the inhibition of G6PD expression and, consequently, breast cancer cell proliferation. Studying the regulation of G6PD by RIP140 in other types of cancers could also be of interest. Furthermore, in addition to their role in cancer metabolism, RIP140 and G6PD are both involved in fatty acid biosynthesis, but also in oxidative stress and inflammatory responses [27,28]. Investigating whether RIP140 regulates G6PD expression in other tissues could be important for diseases related to metabolic disorders, such as obesity, and related diseases, such as diabetes.

## 4. Materials and Methods

### 4.1. Plasmids, siRNA and Reagents

RIP140-expressing vectors (pEFcmyc-RIP140 [29], pEGFP-RIP140 [27]) and control vectors (pEGFP) (Clontech) are described elsewhere. The G6PD-Luc reporter gene containing 1464bp was a gift from Dr. Karadimitris [28]. 6-aminonicotinamide (6AN; #A68203), MTT ((3-(4,5-dimethylthiazol-2-yl)-2,5-diphenyl tetrazolium bromide) (98%, CAS 298-93-1), crystal violet (C0775), G6PDH Activity Assay Kit (MAK015), anti-mouse IgG-FITC antibody (F6257), and monoclonal anti-ß-actin-peroxidase antibody (A3854) were purchased from Sigma-Aldrich (Merck, Darmstadt, Germany). Puromycin (ant-pr-1) was purchased from Invivogen (France). G6PD shRNA (m) (sc-145295-V) was purchased from Santa Cruz Biotechnology (Dallas, TX, USA). Rabbit polyclonal to RIP140 (ab42126) was purchased from Abcam (Cambridge, UK). NADP/NADPH-Glo assay (G9081) was purchased from Promega (Charbonnières-les-Bains, France). Ambion™ Silencer™ Pre-Designed siRNA specific of human G6PD was purchased from Fisher Scientific (#10167104, Illkirch, France).

### 4.2. Cell Culture

Immortalized and transformed mouse embryonic fibroblasts (MEFs) were cultured in F12/Dulbecco’s modified Eagle’s medium supplemented (Gibco, Illkirch, France) with 10% fetal calf serum (Eurobio AbCys, Les Ulis, France) and 1% penicillin/streptomycin (Gibco, Illkirch, France). Primary MEFs were prepared from 13.5-day-old wild-type (WT) or RIPKO mouse embryos [30] and genotyped by PCR. All experiments on mice were performed in accordance with French guidelines (agreement number 201603101538202). We generated immortalized MEFs by following the 3T3 protocol [31] or by infection with retrovirus expressing SV40. Infection with retrovirus-expressing H-RasV12 allowed the transformation of immortalized MEF. Virus production, infection and transfection were performed as previously described [32]. Puromycin (2.5 µg/mL) and hygromycin (65 µg/mL) allowed selection after SV40 and H-RasV12 virus infection, respectively. Immortalized MEFs were transduced with lentiviral particles expressing shRNA against murine G6PD (sc-145295-V) according to the manufacturer’s protocol (Santa Cruz Biotechnology, Dallas, TX, USA). Stable MEFs expressing pEGFP or pEGFP-hRIP140 were described elsewhere [33] and were cultured in the presence of 3.2 µg/mL puromycin. MCF7 were cultured in F12/Dulbecco’s modified Eagle’s medium supplemented with 10% fetal calf serum, 1% penicillin/streptomycin. MDA-M-B436 were cultured in Dulbecco’s modified Eagle’s medium (DMEM) + GlutaMax (Thermo Fisher Scientific, Waltham, MA, USA), supplemented with 10% FBS and 1% penicillin/streptomycin. Breast cancer cell lines were authenticated by short-tandem repeat profiling (Eurofins, Ebersberg, Germany) and all cell lines were tested for mycoplasma contamination.

### 4.3. Real-Time qPCR

Real-time qPCR was conducted as previously described [13]. Briefly, total RNA was extracted from cells using the Quick RNA^TM^ Miniprep kit (Zymo Research, Irvine, CA, USA) according to the manufacturer’s instructions. cDNA was synthetized with 1 µg of RNA and the qScript cDNA master mix (Quanta Bio, Beverly, MA, USA). mRNA expression was determined with a quantitative real-time PCR SYBR Green SensiFAST™ SYBR^®^ No-ROX Kit (Bioline, London, UK) on a Light Cycler^®^ 480 Instrument II (Roche Life Sciences, Penzberg, Germany). Relative expression levels for the mRNAs of interest were normalized to 28S or RS9 housekeeping genes. See Supplemental Tables S1 and S2 for the list of primer sequences.

### 4.4. Protein Detection

Western blot analysis was used to quantify protein expression with the following antibodies: anti-G6PD (1:1000 ab993—Abcam, Cambridge, UK) and anti-Actin HRP (1:10000 A3854—Merck, Darmstadt, Germany).

### 4.5. Luciferase Reporter Assay

For gene reporter assays, cells were plated in 96-well plates and transfected with JetPEI (Polyplus, Illkirch-Graffenstaden, France) according to the manufacturer’s protocol. Firefly luciferase activity was normalized to renilla activity.

### 4.6. Cell Proliferation Analysis

Cell proliferation was assessed by MTT, crystal violet assays, or using the IncuCyte^®^ Live Cell Analysis Imaging System. The cells were seeded into a 96-well plate in the exponential growth phase at a concentration of 1000 cells per 200 µL in complete medium and allowed to adhere for 16–24 h before exposure to different concentrations of 6-aminonicotinamide (6AN). In the case of the MTT assay, the medium was removed and a solution of 0.5 mg/mL MTT (medium as a solvent) was added to each well for 3 h at 37 °C and 5% CO_2_. Formazan crystals were solubilized in dimethylsulfoxide and absorbance was read at 560 nm on a spectrophotometer (Pherastar microplate reader, BMG Labtech, Ortenberg, Germany). In the case of the crystal violet viability assay, the medium was replaced with a 0.5% crystal violet solution (containing 4% formaldehyde, 30% ethanol, 0.17% NaCl) for 15 min at room temperature; then the cells were washed three times with H2O and allowed to dry. A total of 100 µL of 10% acetic acid was added to each well for 20 min with shaking. The absorbance was read at 595 nm on the plate reader (Pherastar microplate reader). For all assays, data were normalized to the cell density at day 1.

### 4.7. Soft-Agar Colony Assay

Transformed MEFs were tested for their ability to form colonies in soft agar. Briefly, a total of 8 × 10^4^ cells were suspended in F12/DMEM supplemented with 10% FBS and 1% penicillin/streptomycin containing 0.4% Noble agar (Sigma Chemical Co., St. Louis, MO, USA) and seeded onto 6-well plates coated with 1% agar in F12/DMEM (10% FBS). The medium was refreshed once a week. At week 4, colonies were stained with 0.5% crystal violet solution, photographed and counted with ImageJ software.

### 4.8. G6PD Activity Assay

Fresh cell culture plates were rinsed with cold PBS and lysed with lysis buffer (100 mM potassium phosphate buffer pH 7.8, 2 mM EDTA, 1 mM dithiothreitol, 1% Triton X-100) supplemented with protease inhibitor cocktails (cOmplete™ Protease Inhibitor Cocktail, Merck). Cells were scrapped and the cell lysate was centrifuged at 12,000× *g* at 4 °C for 15 min. Then, 5 μL of lysate was used for protein determination by the DC Protein Assay (Bio-Rad Laboratories, Hercules, CA, USA). G6PD activity was determined with a Glucose-6-Phosphate Dehydrogenase Assay Kit (MAK015, Merck, Darmstadt, Germany) according to the manufacturer’s instructions using 10 µg of protein. G6PD activity was determined as milliunits/mL, then normalized with respect to the activity in WT MEF samples.

### 4.9. NADP/NADPH Assays

Five thousand MEFs were seeded per well in a 96-well plate in 50 µL of F12/DMEM supplemented with 10% FBS and 1% penicillin/streptomycin. After 24 h, the NADP/NADPH balance was measured by NADP/NADPH-Glo Assay (Promega, Madison, WI, USA) according to the manufacturer’s protocol.

### 4.10. Statistical Analysis

For statistical analysis, nonparametric unpaired two-tailed independent samples Student’s *t*-test was performed using GrapdPad. In all figures, bars represent the mean ± SEM. Statistical analysis was considered significant if a null hypothesis could be rejected when at least *p* < 0.05 (*** *p* < 0.001, ** *p* < 0.01, * *p* < 0.05).

## Data Availability

All data are contained within the article or Supplementary Materials.

## Supplementary Materials

The following supporting information can be downloaded at: https://www.mdpi.com/article/10.3390/ijms23137419/s1. Reference [34] is cited in the supplementary materials.
