# Supplementary material for: The Transcription Coregulator RIP140 Inhibits Cancer Cell Proliferation by Targeting the Pentose Phosphate Pathway
Source: Int J Mol Sci. 2022 Jul 4;23(13):7419. doi: 10.3390/ijms23137419 (PMC9267222; doi:10.3390/ijms23137419)
Supplement: Supplementary file 1 [file ijms-23-07419-s001.zip › ijms-1778338-supplementary.pdf]

International Journal of
Molecular Sciences

MDPI

Article

The Transcription Coregulator RIP140 Inhibits Cancer Cell Proliferation by Targeting the Pentose Phosphate Pathway

Valentin Jacquier [1], Delphine Gitenay [2], Vincent Cavaillès [1,†] and Catherine Teyssier [1,*,†]

1 IRCM, Institut de Recherche en Cancérologie de Montpellier, INSERM U1194, University of Montpellier, 34298 Montpellier, France; valentinjacquier1993@gmail.com (V.J.); vincent.cavailles@inserm.fr (V.C.)
2 IRMB, University of Montpellier, INSERM, 34295 Montpellier, France; delphine.gitenay@inserm.fr (D.G.)
* Correspondence: catherine.teyssier@inserm.fr
† These authors contributed equally to this work.

Supplemental informations

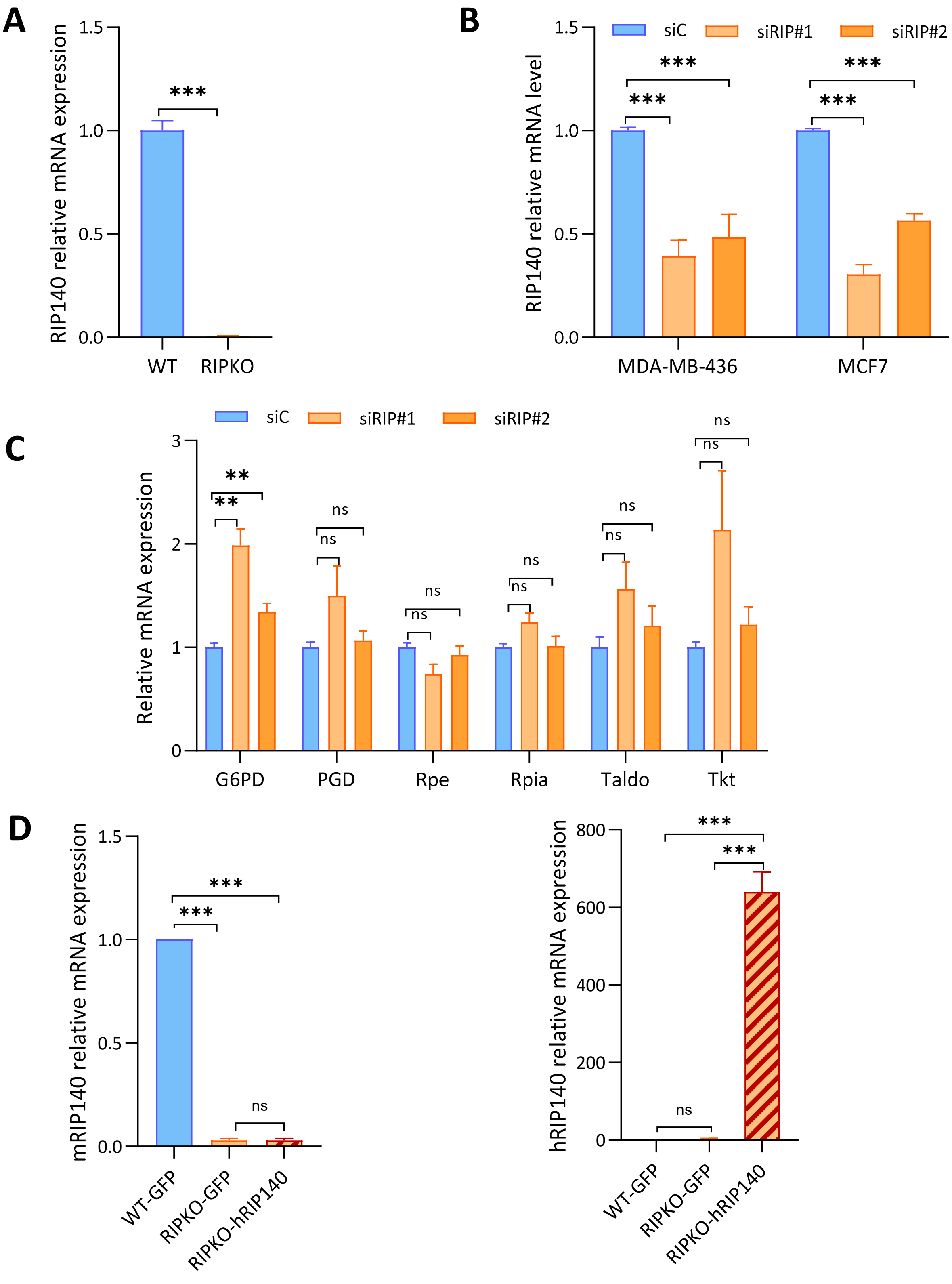

Figure S1. RIP14O inhibits G6PD gene expression at the transcriptional level. (**A**) RIP140 mRNA expression was assessed by RT-qPCR in MEF WT or RIP140 knock-out (RIPKO). The values are normalized to WT samples. (**B**) RIP140 mRNA expression was assessed by RT-qPCR in MCF7 and MDA-MB-436 cells transfected with control

siRNA (siC) or RIP140 siRNAs (siRIP#1; siRIP#2). Values are normalized to control siRNA samples. (**C**) mRNA expression of the indicated genes was assessed by RT-qPCR in MCF7 cells transfected with control siRNA (siC) or RIP140 siRNAs (siRIP#1; siRIP#2). Values are normalized to control siRNA samples. (**D**) Murin (left panel) and human (right panel) RIP140 mRNA expression were assessed by RT-qPCR in MEF WT or RIP140 knock-out (RIPKO) overexpressing GFP or human RIP140 (hRIP140). The values are normalized to WT-GFP samples. All experiments were done at least three times. Values are means ± SEM; **p <0.01, ***p <0.001, ns = not significant.

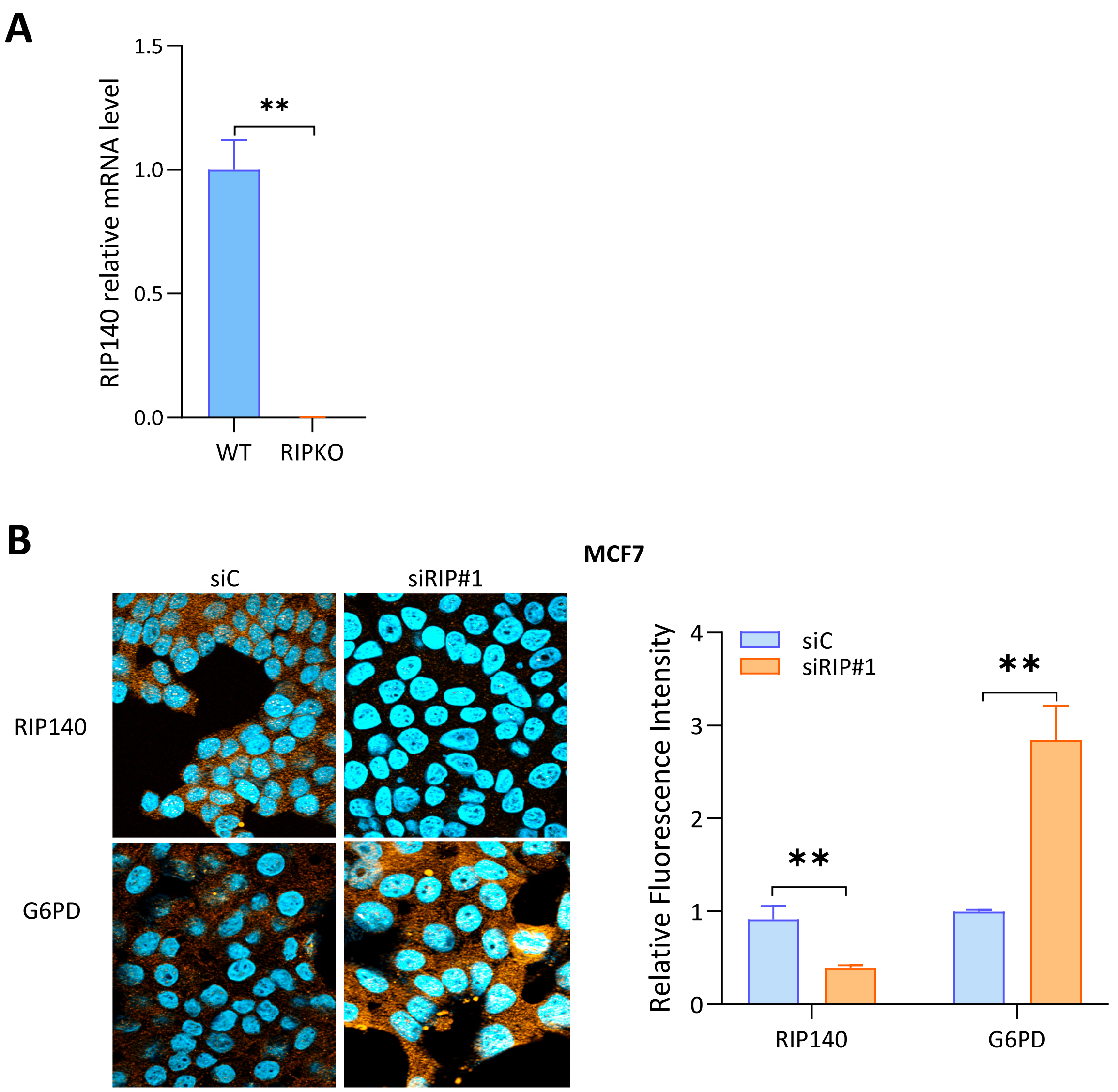

Figure S2. RIP140-deficiency increases G6PD activity. (**A**) RIP140 mRNA expression was assessed by RT-qPCR in MEF WT or RIP140 knock-out (RIPKO). The values are normalized to WT samples. Values are means ± SEM of at least three independent experiments; **p <0.01. (**B**) RIP140 and G6PD protein level was assessed by immunofluorescence using anti-RIP140 and anti-G6PD antibodies in MCF7 cells transfected with control siRNA (siC) or RIP140 siRNAs (siRIP#1). Hoechst 33342 was used for nuclei detection (left panel). Fluorescence intensity was quantified using Image J software in 6 different fields randomly chosen for each condition. Values are normalized to that of control siRNA. Values are means ± SEM; **p <0.01 (right panel).

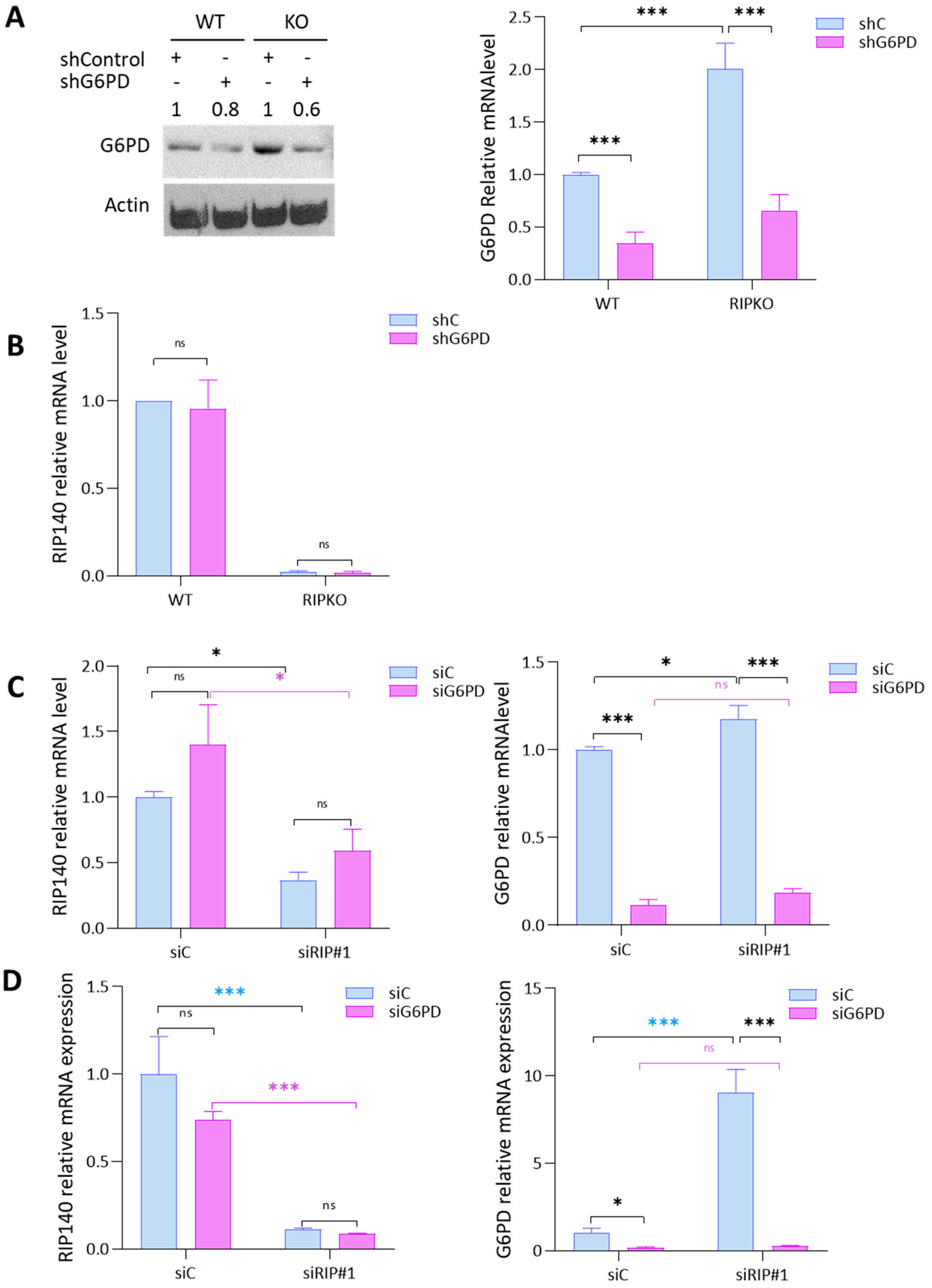
A
WT
KO
shControl
shG6PD
1
0.8
1
0.6
G6PD
Actin
G6PD Relative mRNAlevel
shC
shG6PD
WT
RIPKO
B
RIP140 relative mRNA level
shC
shG6PD
ns
WT
RIPKO
C
RIP140 relative mRNA level
siC
siG6PD
siRIP#1
G6PD relative mRNAlevel
D
RIP140 relative mRNA expression
G6PD relative mRNA expression

Figure S3. G6PD is required for the proliferative advantage of RIP140-deficient cells. (**A**) G6PD expression level was assessed by Western blot (left panel) quantified by Image J software and by RT-qPCR (right panel) in MEF transfected with shRNA control or shG6PD expressing lentivirus. (**B**) RIP140 expression level quantified by RT-qPCR in MEF used in Figure 3. (**C-D**) siRNA efficiency was verified by RT-qPCR for RIP140 (left panel) and G6DP (right panel) 48h after transfection in MDA-MD-436 (**C**) and in MCF7 (**D**) cells used in Figure 3C. Data are presented as the mean ± SEM of at least three independent experiments. The statistical significance of differences between groups was evaluated via one-way analysis of variance (ANOVA), followed by Tukey's multiple-comparison test, using the GraphPad Prism software. *p <0.05, ***p <0.001 , ns = not significant.

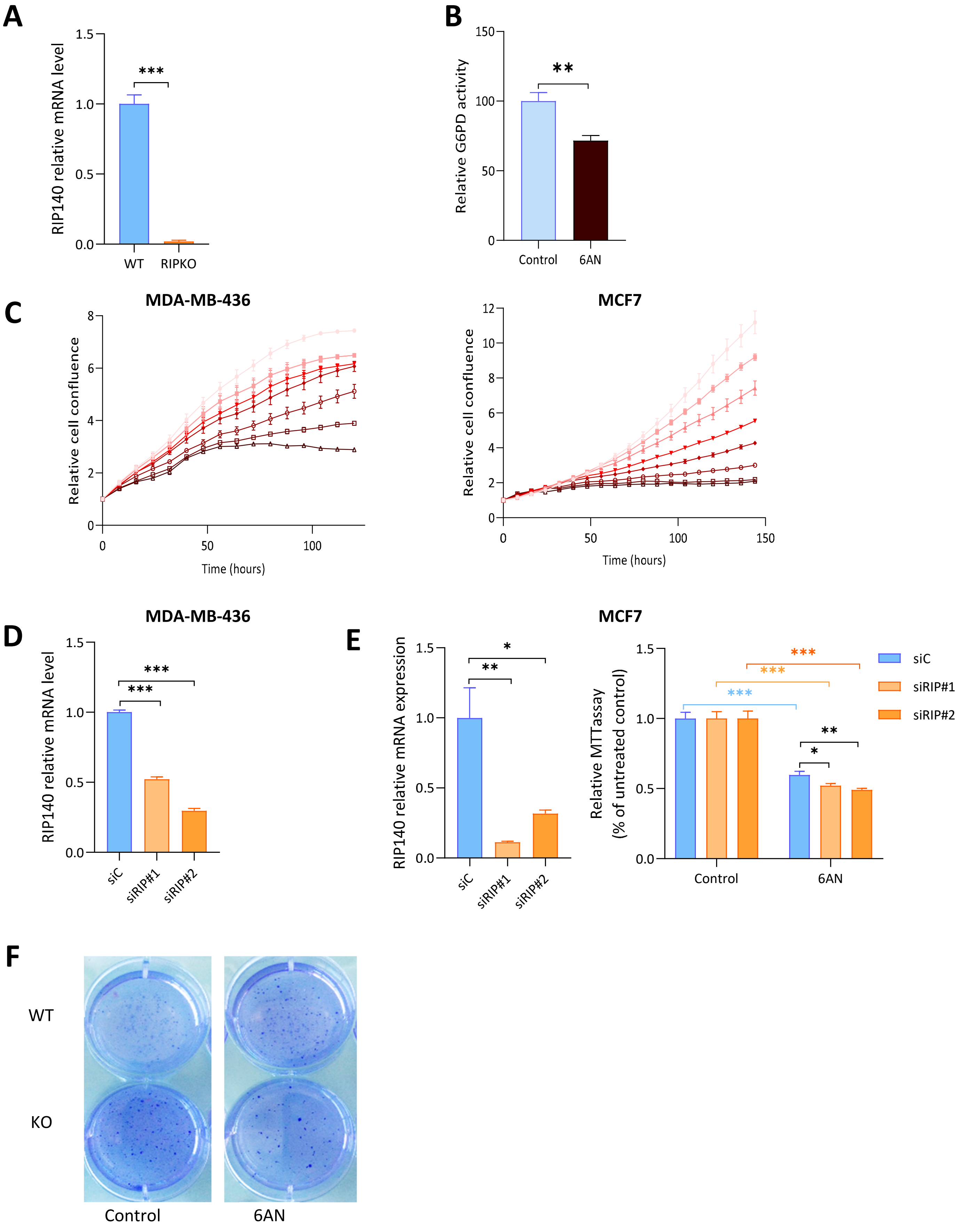
A
RIP140 relative mRNA level
***
WT
RIPKO
B
Relative G6PD activity
**
Control
6AN
C
MDA-MB-436
Relative cell confluence
Time (hours)
MCF7
Relative cell confluence
Time (hours)
D
MDA-MB-436
RIP140 relative mRNA level
***
***
siC
siRIP#1
siRIP#2
E
MCF7
RIP140 relative mRNA expression
*
**
siC
siRIP#1
siRIP#2
Relative MTTassay
(% of untreated control)
siC
siRIP#1
siRIP#2
Control
6AN
F
WT
KO
Control
6AN

Figure S4. RIP140-deficiency sensitizes cells to G6PD-inhibition. (**A**) RIP140 mRNA expression was assessed by RT-qPCR in MEF WT or RIP140 knock-out (RIPKO). The values are normalized to WT samples. (**B**) The effect of 6-aminonicotinamide (6-AN, 10μM) on G6PD enzymatic activity was evaluated in MEF WT samples. Values are normalized to protein content and to that of untreated samples. (**C**) To fix the 6-AN concentration to be used in breast cancer cells, cell proliferation of MDA-MD-436 (left panel) and MCF7 (right panel) cells was assessed using the IncuCyte® Live Cell Analysis Imaging System in a 96-well plate with 2000 cells per well for the indicated times. Cells were treated at day 1 with increasing doses of 6-AN (0, 1, 2, 5, 10, 20, 50, 100μM). Values are normalized to that of untreated samples at day 1. 20μM of 6-AN inhibited cell proliferation about 70% in both breast cancer cell lines and was used in the subsequent experiments. (**D**) RIP140 mRNA expression in MDA-MB-436 cells transfected with control siRNA (siC) or RIP140 siRNAs (siRIP#1; siRIP#2). Values are normalized to control siRNA samples. (**E**) Right panel: RIP140 mRNA expression in MCF7 cells transfected with control siRNA (siC) or RIP140 siRNAs (siRIP#1; siRIP#2). Values are normalized to control siRNA samples. Left panel: MTT assay in MCF7 cells transfected with control siRNA (siC) or RIP140 siRNA (siRIP#1, siRIP#2) and treated for seven days with 6AN (20μM). Values are normalized to that of untreated control siRNA (Right panel). (**E**) Crystal violet staining of soft agar colonies quantified in Figure 4E. All experiments were done at least three times. Values are means ± SEM; *p <0.05, **p <0.01, ***p <0.001, ns = not significant.

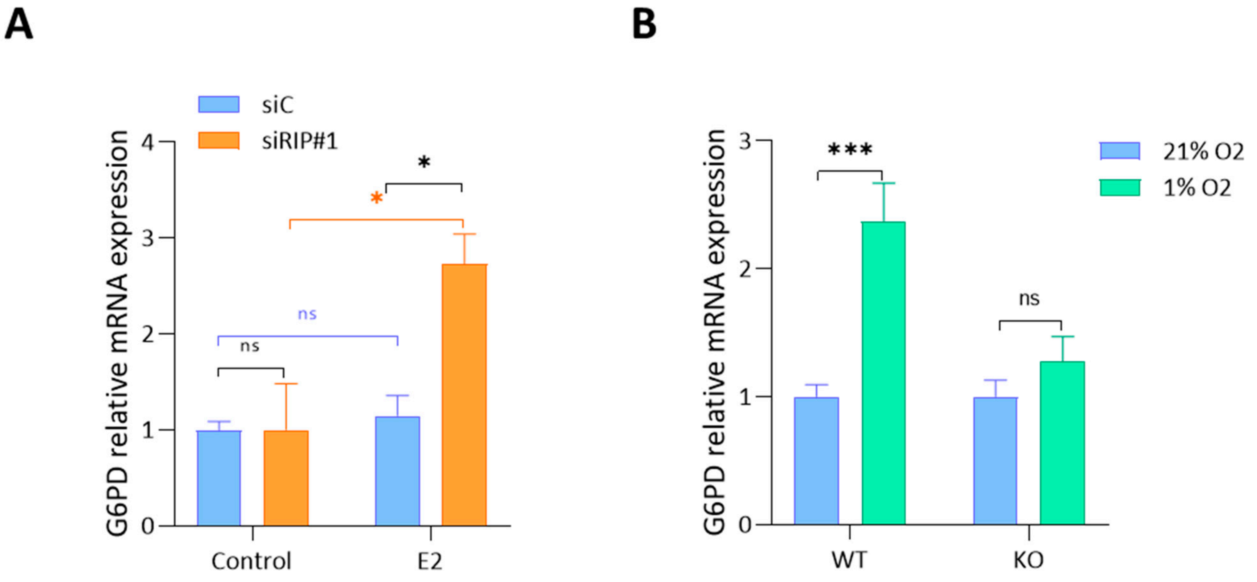

Figure S5. Regulation of G6PD expression by RIP140 under estrogenic and hypoxia conditions. (**A**) G6PD mRNA expression was assessed by RT-qPCR in MCF7 cells transfected with control siRNA (siC) or RIP140 siRNA (siRIP#1) and treated for 24h with Estradiol (E2 10E-8M) . The values are normalized to untreated siC samples. (**B**) G6PD mRNA expression was assessed by RT-qPCR in MEF WT or RIP140 knock-out (RIPKO) exposed to hypoxia (1% O2) for 24h. The values are normalized to that of samples under normoxia (21% O2). All experiments were done at least three times. Values are means ± SEM; *p <0.05, ***p <0.001, ns = not significant.

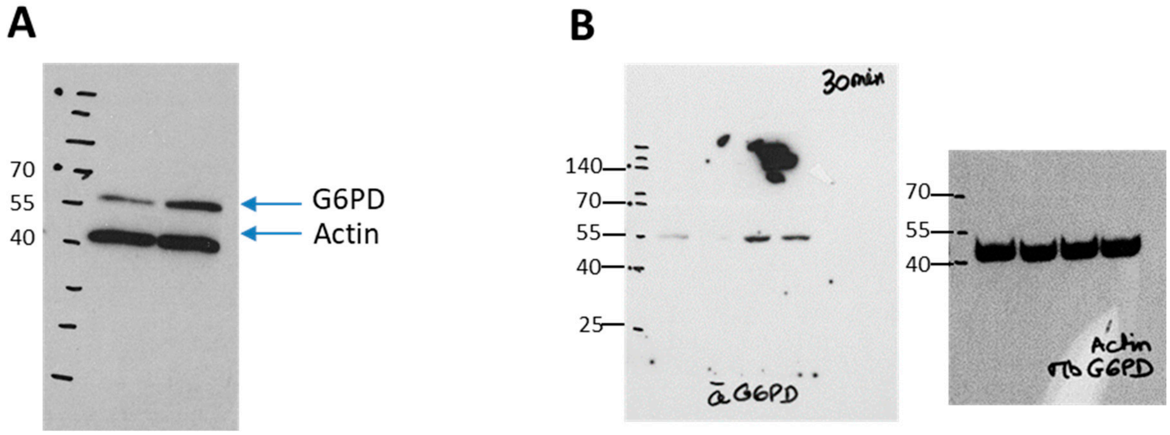

Figure S6. Unprocessed original scans of blots. Unprocessed images of Western blots used in Figure 2 (**A**) and in Figure 3 (**B**). Molecular size markers in kDa.

Supplemental Materials and Methods

Immunofluoresence. Cells grown on glass coverslips were fixed with 3,7% Formaldehyde for 10 minutes, then whashed with 0,05% Tween-20-PBS, permeabilized with 1% Triton-X100-PBS for 10 minutes followed by a wash with 0,05% Tween-20-PBS. The slides were then blocked with 1% bovine serum albumin for 3 hours, before applying primary antibodies overnight. The primary antibodies used: RIP140 antibody (1:100, Ab42126; Abcam Cambridge, UK), G6PD antibody (1:200, ab993; Abcam, Cambridge, UK). The secondary antibody used was AlexaFluor555-conjugated goat anti-rabbit (1:250, #A27039, Thermo Fisher Scientific, Whaltham, USA). The cells were counterstained with Hoechst 33342 (Merck, Darmstadt, Germany) and analyzed with a AxioVision Microscope (Zeiss, Lena, Germany).

IncuCyte® Live Cell Analysis. Cell proliferation was directly determined and imaged using IncuCyte® Live Cell Analysis Imaging System[1] (Essen BioScience Co., Ltd.,Ann Arbor, Michigan, USA). Briefly, 2000 cells were plated into a 96-well plate and put into the IncuCyte® 24 hours later. Scans were scheduled every 8 hours using Phase Image channel and 4x magnification for 7 days. Scans were retrieved using the Analysis Imaging System measuring classic confluence.

Table D1. Murine primer sequences.

m-G6pd2-FOR	GGC TTC TTG GTC ATC ATC GT
m-G6pd2-REV	TCC GAG ATA TAC CAG GCG AC
mPgd-FOR	CATCGATGATGATGTCACCC
mPgd-REV	GGGTCATCCTGCTTGTGAAG
mRpia-FOR	CACTTCCAATTCCCAGCACT
mRpia-REV	CCTCCACGATGTCCAAGG
mRpe-FOR	CACAGCCATTGGTTTTACCC
mRpe-REV	GGTCACCCTGTGGTTGAAAG
mTkt-FOR	ATGGAAAAACAGGACAGCCA
mTkt-REV	GCATCAGCTCCATCCAGG
mTaldo1-FOR	CTTGGGGCTTGTATTCATCG
mTaldo1-REV	GGTAAAGCGCCAGAGGATG
mRipP140-FOR	AGAACGCACATCAGGTGGCA
mRip140-REV	GATGGCCAGACACCCCTTTG
RS9-FOR	CGGCCCGGGAGCTGTTGACG
RS9-REV	CTGCTTGCGGACCCTAATGTGACG

Table S2. Human primer sequences.

h-G6PDH-FOR	CAC CAG ATG GTG GGG TAG AT
h-G6PDH-REV	AGA GCT TTT CCA GGG CGA
hPGD-FOR	GCCTTGGAAGATGGTCTTGA
hPGD-REV	GTCAGTGGTGGAGAGGAAGG
hRPIA-FOR	CGATCCAGATCACTGAGGGT
hRPIA-REV	GCTGAAAGGGTGAAGCAAGA
hRPE-FOR	GGCTTTACCCACTGTTCTGG
hRPE-REV	GGATGCTAGACTCTGGGGC
hTKT-FOR	GCATGGTGTGGAAAAAGAGG
hTKT-REV	CGCCTACGTATCAGCTCCA
hTALDO1-FOR	ATCCTGGGGCTTGTACTCGT
hTALDO1-REV	GAAGCGTCAGAGGATGGAGT
hRIP140-FOR	AATGTGCACTTGAGCCATGATG
hRIP140-REV	TCGGACACTGGTAAGGCAGG
28S-FOR	CGATCCATCATCCGCAATG
28S-REV	AGCCAAGCTCAGCGCAAC

Table S3. siRNA sequences.

Name	sequence
siControl	(UAAUGUAUUGGAACGCAUA)TT
siRIP140#1	(GAAGCGUGCUAACGAUAAA)TT
siRIP140#2	(AUACGAAUCUUCCUGAUGU)TT

Reference

1. Quantitative Measurement of Cancer Cell Proliferation Using CellPlayer™ Kinetic Proliferation Assay Available online: https://www.news-medical.net/whitepaper/20161003/Quantitative-Measurement-of-Cancer-Cell-Proliferation-Using-CellPlayer-Kinetic-Proliferation-Assay.aspx (accessed on 24 June 2022).
